# Acute Neurological Care in the COVID-19 Era: The Pandemic Health System **RE**silience **PROGRAM** (**REPROGRAM**) Consortium Pathway

**DOI:** 10.3389/fneur.2020.00579

**Published:** 2020-05-29

**Authors:** Sonu Bhaskar, Divyansh Sharma, Antony H. Walker, Mark McDonald, Bella Huasen, Abilash Haridas, Manoj Kumar Mahata, Pascal Jabbour

**Affiliations:** ^1^Pandemic Health System REsilience PROGRAM (REPROGRAM) Consortium, REPROGRAM Acute Care Sub-committee^†^, Sydney, NSW, Australia; ^2^Department of Neurology, Liverpool Hospital, Liverpool, NSW, Australia; ^3^Neurovascular Imaging Laboratory & NSW Brain Clot Bank, Ingham Institute for Applied Medical Research, Liverpool, NSW, Australia; ^4^The University of New South Wales Sydney, UNSW Medicine, Sydney, NSW, Australia; ^5^The University of New South Wales Sydney, UNSW Medicine, Sydney, NSW, Australia; ^6^Lancashire Cardiac Centre, Blackpool Victoria Hospital, NHS, Lancashire, United Kingdom; ^7^Department of Neurology, University of Virginia, Charlottesville, VA, United States; ^8^Department of Interventional Neuroradiology, Lancashire University Teaching Hospitals, Preston, United Kingdom; ^9^Pediatric Neurosurgery, Cerebrovascular and Skull Base Neurosurgery, St Joseph's Hospital, Tampa, FL, United States; ^10^Department of Stroke and Neurointervention, Woodlands Multispeciality Hospital Limited, Kolkata, India; ^11^Division of Neurovascular Surgery and Endovascular Neurosurgery, Thomas Jefferson University and Jefferson Hospital for Neuroscience, Philadelphia, PA, United States

**Keywords:** Coronavirus disease 2019 (COVID-19), Personal Protective Equipment (PPE), safety, acute stroke, guidelines, reperfusion, neurointervention, surgery

## Abstract

The management of acute neurological conditions, particularly acute ischemic stroke, in the context of Coronavirus disease 2019 (COVID-19), is of importance, considering the risk of infection to the healthcare workers and patients and emerging evidence of the neuroinvasive potential of the virus. There are variations in expert guidelines further complicating the picture for clinicians in acute settings. In this light, there is a compelling need for further formulation of recommendations that compile these variations seen in the numerous guidelines present. Health system protocols for managing ongoing acute neurological care and intervention need consideration of safety and well-being of the frontline healthcare workers and the patients. We examine existing pathways and their efficacy to mitigate viral exposure to the healthcare workers and patients and synthesize a systemic approach to manage patients with acute neurological conditions in the COVID-19 scenario. Early experiences with a COVID-19 positive stroke patient treated with endovascular thrombectomy is presented to highlight the urgent need for adequate personal protective equipment (PPE) during acute neuro-interventional procedures.

## Introduction

Neurotropism is a well-known feature of beta-coronaviruses, of which severe acute respiratory syndrome coronavirus 2 (SARS-CoV-2), the virus which causes Coronavirus Disease 2019 (COVID-19) (1), is one, with effects on the brain stem, and in particular, the cardiorespiratory center thought to result in breathing dysfunction (2). The Italian experience has displayed the presence of neurological symptoms in COVID-19 positive patients (3). The Chinese study from Wuhan published in JAMA Neurology reported neurological manifestations in a significant proportion (36.4%) of patients with COVID-19 (4). Recent findings surrounding anosmia as an early symptom of COVID-19 have invoked further interest in this hypothesis (5). The role of the central component in hyposmia could also be suspected. Those presenting with symptoms of skeletal muscle damage are at higher risk of liver and kidney damage. It is evident that the virus is able to cross the blood-brain barrier (BBB), which is postulated to occur post-infection due to interactions with the Angiotensin-Converting Enzyme 2 (ACE2) receptor present at various sites within the cerebral circulation (6). Another case report on a female airline worker with COVID-19 positive status developing acute necrotizing hemorrhagic encephalopathy (7), a condition that is typically seen following cytokine storm in influenza, suggests possible BBB compromise. Independent of possible neurotropism, COVID-19 infection is associated with coagulopathy (elevated D-dimer and severe platelet reduction) and may disrupt blood pressure regulation through interaction with the ACE2 receptor. COVID-19 could possibly contribute to ischemic and hemorrhagic stroke aside from neurotropism (8). Taken together these anecdotal reports suggest a possible neuroinvasive potential of the virus.

## Acute Ischemic Stroke

Management of patients with acute ischemic stroke during COVID-19 pandemic could be challenging and certain precautions must be taken in order to protect healthcare workers, particularly in the delivery of endovascular treatment, where aerosol could be produced during the procedures, to prevent further vector transmission (9). As a result of this, various modifications of the traditional code stroke are being discussed amongst hospitals, and in particular, Khosravani et al. (10) propose the concept of the “Protected Code Stroke” whereby management of patients with a suspected stroke is modified in the context of the COVID-19 pandemic to protect healthcare workers. A conservative approach involving fever screening, history taking to rule out COVID-19 risks and the presence of infectious symptoms could replace routine “Code Stroke.”

Minimizing healthcare workers in the same room as the patient, specifications surrounding personal protective equipment use, and the delegation of specific roles to limit the risk of infection have been suggested. However, this protocol is not ratified by other major associations and does not consider the surgical aspects associated with endovascular treatment, a major gap that must be addressed.

Various bodies have put forth guidelines into how surgery should be conducted in these times to minimize harm to patients and healthcare workers alike. However, they are non-specific to endovascular treatment. Nonetheless, general Intercollegiate Surgical Guidelines (11) are available, and emphasize the importance of not undertaking procedures that may result in poorly controlled aerosol production, minimization of theater staff, team changes required during a prolonged surgery, and intubation and extubation within the operation theater itself, with only necessary staff members present. This differs from the “*Society of American Gastrointestinal and Endoscopic Surgeons and The European Association of Endoscopic Surgery Recommendations Regarding Surgical Response to COVID-19 Crisis* (12),” which recommend that “unless there is an emergency, there should be no exchange of room staff.”

Notably, neither of these guidelines are specific to endovascular treatment. The *Society of Neurointerventional Surgery* recently released “recommendations for the care of emergent neuro-interventional patients in the setting of COVID-19 (13),” which consider the management of patients before, during and after thrombectomy. They agree with the model proposed by Khosravani et al. (10) with regards to presuming COVID positive status unless proven otherwise. Notably, these guidelines concur with the “*Consensus Statement from Society for Neuroscience in Anesthesiology & Critical Care”* about “*Anesthetic Management of Endovascular Treatment of Acute Ischemic Stroke During COVID-19 Pandemic* (9)*,”* in that general anesthesia should be used if there are concerns surrounding the need for mid-procedural conversion and intubation which could be very detrimental and could expose the whole team, a scenario that should be avoided at all cost. However, these latter guidelines do not address the issue of separating COVID-19 patients from others in terms of scanning equipment, radiology suites, and decontamination protocols.

## Recommendations for Treating Patients With Neurological Symptoms and Suspected Acute Ischemic Stroke Patients

**Figure 1:**
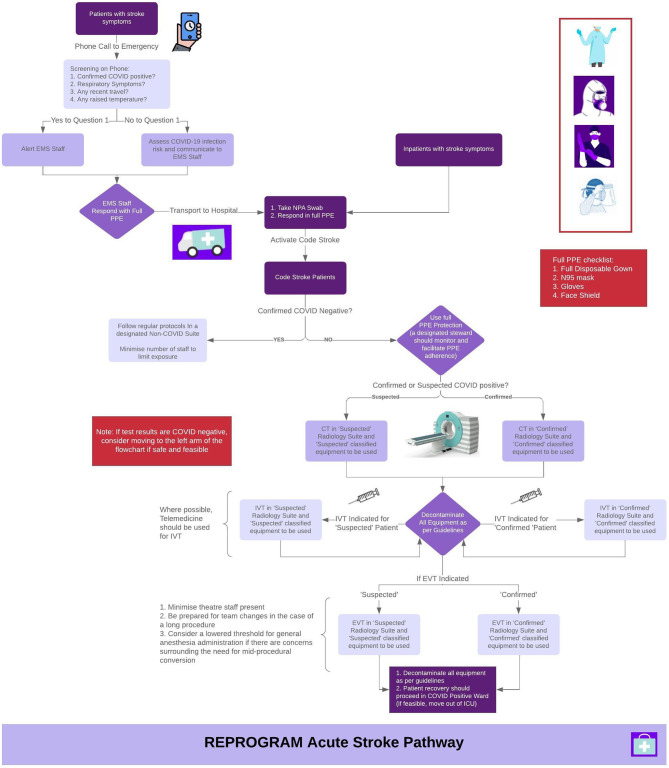
Proposed acute stroke pathway in the setting of the coronavirus 2019 (COVID-19) pandemic. Notably, there are 3 different suite options recommended: Non-COVID, Suspected COVID and Confirmed COVID. EMS, Emergency Medical Services; NPA, Nasopharyngeal Aspirate; PPE, Personal Protective Equipment; IVT, Intravenous Thrombolysis; EVT, Endovascular Therapy; ICU, Intensive Care Unit; CT, Computed Tomography.

Given the possible neuroinvasive potential of COVID-19, there is a need to consider both the short and long-term implications of COVID-19, and implement systems-level methods of assessing, addressing, and longer-term monitoring (Figure 1). We expect that there is a significant amount of variability based on institution and country with respect to COVID-19 testing. For example, the earliest possible result time for COVID testing at one of our hospitals is 7 h but the serology test that would take minutes to give a result was just Food and Drug Administration (FDA) approved and hopefully will be introduced soon but until this is available widely it will be practically difficult to rule out COVID-19 during code stroke (at least at many hospitals in the US and elsewhere), and as such, we propose that all patients undergoing code stroke be presumed COVID-19 positive. This is concurrent with the American Heart Association (AHA) emergency guidelines for stroke centers in the context of COVID-19 (14). All COVID-19 positive patients should be triaged into COVID-19 neuro or COVID-19 non-neuro wards depending upon the presence of neurological symptoms (6). Common neurological complaints include dizziness, headache, anosmia, and dysgeusia (14).

In patients with a suspected acute stroke:

- All acute stroke patients should be treated as COVID positive until proven otherwise, and full Personal Protective Equipment (PPE) should be used when responding to a code stroke (10, 13).
- Telemedicine should be used to determine eligibility and perform intravenous thrombolysis [trans plasminogen activator (tPA)] to minimize potential exposure to infectious patients (14, 15). Patients who receive tPA do not need to be admitted to the ICU, if stable. Prior to the pandemic, it was standard practice in the US to admit all post-tPA patients to the ICU for 24 h. However, the AHA recommends that there is little evidence to support post-tPA ICU stay (14).
- Separate scanning equipment and radiology suites for negative, suspected, and confirmed COVID-19 patients, with clear decontamination protocols after each patient (16).
- Separate suites for endovascular treatment of negative and suspected/confirmed COVID-19 patients, with extra equipment stocked in the latter to prevent staff having to retrieve equipment. Clear decontamination protocols after each patient (13).
- In all theaters, minimize exposure to staff and the number of perioperative workers (10, 11).
- In the case of long procedures, team changes should be encouraged to minimize prolonged exposure to healthcare workers (11).
- A lowered threshold for general anesthesia administration in terms of concerns surrounding the need for mid-procedural conversion (9, 13).
- Where possible, post thrombectomy recovery should occur outside of ICU in the stroke unit if those beds are required for COVID-19 patients (14).
- It is recommended that suspected COVID-19 patients should be treated as COVID-19 positive until the polymerase chain reaction (PCR) diagnosis confirms otherwise, and such patients should be admitted to COVID-19 positive wards. Separate stroke units for COVID-19 positive and negative patients are recommended.
- To ensure the quality of stroke care for COVID-19 stroke patients, such patients could be admitted to other wards for COVID-19 positive patients. Dysphagia management, physical or logo therapy, and standard in-hospital rehabilitation of stroke patients should be provided; however, concerned staff should wear adequate PPE to prevent exposure and transmission.
- Healthcare workers in secondary hospitals and radiology facilities are recommended to wear adequate PPEs when caring for someone with a confirmed or suspected case of COVID-19.
  It is advised that patients in which neurological symptoms are present:
- Patients should be monitored for short-term and/or possibly long term cognitive or neurological impairments. Cognitive impairment could be assessed using routine tests such as Mini-Mental State Examination (MMSE) by treating clinicians. Large scale community screening with good sensitivity/specificity could also be administered using telephone, by informant proxy or directly by post [such as Cognitive Assessment Screening Test (CAST)] provided the test has a good sensitivity/specificity balance (>85%) (17).
- For patients presenting with neurological symptoms in future, past COVID-19 infection should be ascertained, along with the clinical severity, and corroborating imaging findings.
- In addition, imaging could be used to assess the damage to the blood brain barrier (BBB) to examine whether COVID-19 induces a transient or long-term change. BBB assessment and permeability quantification could be done either: (a) semi-quantitatively by comparing the scans before and after contrast injection, or (b) quantitatively using perfusion-weighted or permeability magnetic resonance imaging (MRI) technique, vis a vis dynamic contrast-enhanced MRI (DCE-MRI) (18).

## Other Acute Neurological Conditions

For all acute neurological conditions, a major concern revolves around the decrease in the proportion of acute presentations due to fear of contracting COVID-19 while accessing health services and the presumption that all healthcare resources are now mobilized to prioritize COVID-19 patients (14). This could have negative consequences vis a vis long-term disability subsequent to permanent brain damage due to acute neurological emergencies such as traumatic brain injury (19). Similarly, earlier symptoms of emergent brain tumors, such as headache and ataxia (20) may be neglected or cranial neuropathies from mass effect of a brain aneurysm, due to the perceived cons of seeking help. As of yet, significant gaps exist in the literature pertaining to how to address delayed or absence of presentation. Use of telemedicine where possible, social distancing within clinics for patients coming to the hospitals and systems-level separation of patients with fever and respiratory symptoms from those without having been proposed as possible solutions to minimize the impact (21).

Public health campaigns surrounding measures that are in place to minimize infection transmission and ill consequences of failing to present with a condition that does indeed warrant medical attention need to be pursued. Also, the long-term negative impact of the delayed presentation should be emphasized. A recent case report identified a link between frequent convulsive seizures and COVID-19 infection in the context of emergent epilepsy (22). In light of these anecdotal findings, it is relevant that guidelines pertaining to seizure management in COVID-19 cases are not available, to the best of our knowledge. With regards to chronic epilepsy patients, longer-term medicine prescription, use of telemedicine, and optimal seizure management plans have been recommended (23). Similar issues exist with respect to the management of aneurysmal presentations as no specific guidelines exist in the COVID-19 scenario.

The number of COVID 19 positive patients under 18 years of age represent 1.7% of total lab-confirmed cases in the USA (24). Given the relatively low proportion of COVID-19 pediatric patients, neurological manifestations are very unlikely to be delineated.

## Supporting Our Healthcare Workers

This pandemic is adversely challenging the health systems, causing stress, fear to healthcare workers, with the pressures of lengthened hours, lack of PPE equipment and systemic changes that are having to be implemented to protect them (10, 14). Indeed many healthcare workers have expressed publicly in the media and on social media channels that the risk of infecting their families is a source of constant stress to them and impacting their intimate relationships significantly (25). Indeed it is also overlooked that the scarcity of resources can impact the management of patients and potentially result in some patient who may have ordinarily fared better having worse outcomes, another key factor in terms of mental health issues and also indeed the morale of healthcare workers, which can have longer terms impacts in terms of the efficiency and drive of health systems (26).

Considering public health ethics, and more specifically the concept of utilitarianism which forms a key part of this, the need to protect our frontline healthcare workers and support their health becomes evident. Utilitarianism refers to judging actions based on how much good they will do for the greatest number of people - thereby forming the backbone of ethics and health policy debate underpinning the crisis (27). Protecting our healthcare workers gives the most benefit. This can, therefore, involve protecting them from contracting the infection, which could then be spread to their families, other patients, and resultantly the community, as well as focusing on their psychological health so they are able to discharge their duties efficiently and effectively. Various strategies have been proposed for addressing these issues.

### Training

It is pivotal that any changes to protocols, such as those related to changes in how to carry out code stroke actions are well-rehearsed, which may include simulation training with the revised protocol (10). An extra healthcare worker on the team will be needed to observe the team while at work to try to detect any breach in the COVID-19 precaution protocols and at the end of a procedure to help undress the team and clean their PPEs.

### Breaks

Managing a pandemic of this proportion can undeniably cause stress and fear. As such it has been proposed that healthcare workers, particularly those working with COVID-19 positive cases, be given regular breaks (16) and encouraged to recognize their limits (28). We also propose that healthcare workers be given information pertaining to relaxation and coping strategies; whilst many healthcare workers may already be aware of these, a reminder may be beneficial.

### Team Cohesion and Peer Support

The World Health Organization “Mental health and psychosocial considerations during the COVID-19 outbreak” document advocates the role of a “buddy” or peer support system for more experienced clinicians to assist and support their less experienced colleagues, as a means to not only help manage stress but also learn how to efficiently enact the protocols that may be in place in an organization (29). This is especially relevant as the health systems are being reorganized and protocols are being revised regularly, sometimes on a daily basis (30). Online peer-support networks for discussions as well as social media and messaging chat groups may provide a valuable outlet for clinicians.

### Supporting Home Environments

Planning how healthcare workers will interact with their families and reorganize their living arrangements can help de-escalate the stressors as reported in the media (25). The Victorian government in Australia has announced that all healthcare workers required to self-isolate or tested positive for COVID-19 will be provided hotel accommodation to minimize risks to them and to their families, with an indication to expand this model to other states and territories (31). It is important for these recommendations to be specific to avoid creating further anxiety among healthcare workers (32).

## Conclusions

**Figure 2:**
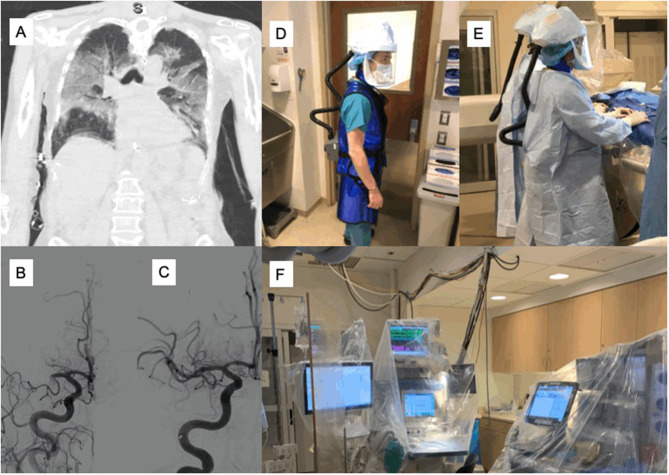
Illustration of an acute stroke reperfusion work-up in a COVID-19 positive case. A patient in the age-group of 65–75 years, with a history of atrial fibrillation and on anticoagulation, presented to an outside facility with difficulty breathing (dyspnea), high temperature and severe cough. COVID-19 work-up was followed and the patient tested positive for COVID-19. Chest computed tomography (CT) revealed bilateral infiltrates **(A)**. The patient was intubated and sedated a day later. On Day 3, the nurse noticed that she was not moving the left side to painful stimuli; given the time of onset could not be determined (unknown), intravenous thrombolysis was not given. Baseline non-contrast computed tomography (NCCT) head was normal; the patient was transferred to the comprehensive stroke center. CT angiography (CTA) showed the right M1 and A2 occlusions. Pre-intervention digital subtraction angiography (DSA) confirmed occlusion in the right M1 and A2 arteries **(B)**. Endovascular thrombectomy (EVT) was performed successfully with complete angiographic reperfusion (thrombolysis in cerebral infarction (TICI) score of 3). The patient was still intubated by the time the manuscript was written. **(C)** Post-intervention DSA imaging demonstrated good reperfusion outcome (TICI3). A clinician with *3M*™ *Versaflo*™ *TR-600* Powered Air Purifying Respirator (PAPr) – personal protective equipment (PPE) for protection against the air-borne virus is shown **(D)**. The interventional neuroradiology (INR) team doing the EVT procedure while wearing their full PPEs (sterile gown, gloves and PAPr) is shown **(E)**. Post-procedure, all INR suite equipment including anesthesia machines and pyxis are secured using surgical drapes and equipment covers **(F)**. COVID-19, Coronavirus 2019; NCCT, Non-contrast computed tomography angiography; CTA, CT angiography; EVT, Endovascular thrombectomy; TICI, Thrombolysis in cerebral infarction score; DSA, Digital subtraction angiography; PAPr, Powered Air Purifying Respirator; PPE, Personal protective equipment; INR, Interventional neuroradiology.

In the COVID-19 pandemic, acute neurological care is increasingly under stress due to ongoing reorganization and rationing of services to meet the demands of frontline COVID-19 cases. In this article, we have identified and proposed various considerations that may minimize the risk to health systems, healthcare workers, and the patients. The differential diagnosis of severe acute respiratory syndrome CoV (SARS-CoV2) infection should be considered in patients with neurological symptoms during the COVID-19 period (4). This is important to avoid missed or delayed diagnosis and prevent viral transmission. All patients amidst this pandemic should be screened for COVID-19 and telemedicine could be used to triage these patients and possibly deliver intravenous thrombolysis. For those who may be candidates for endovascular thrombectomy, extra precautions need to be taken to minimize procedural risks associated with the aerosol transmission of the COVID-19 virus and possible exposure to the healthcare staff. An example of reperfusion therapy work-up with PPEs in a COVID-19 stroke patient is illustrated in Figure 2. Public health campaigns to educate and increase awareness of the community about the need to seek urgent medical attention should acute neurological symptoms occur. Special considerations also apply for patients with traumatic brain injury and those requiring urgent aneurysm surgery or carotid endarterectomy. We are alarmed at the rising deaths of healthcare workers who are waging a war against the COVID-19 without the provision of adequate PPE to defend themselves. The cost of adopting the proposed protocol and its impact on the quality of care merits further study. The current consortium is expeditiously working toward rapid adoption of the proposed protocol. Further study on the impact and cost these measures may have on the quality of care and its results are envisaged. However, given the nature of the pandemic and emerging situation, the safety of healthcare workers' is paramount and thus justifies the heightened safety measures suggested in our protocol with an anticipation that this would hopefully limit the exposure. Minimizing the harm to healthcare workers should be a priority as potential exposure can not only compromise the health systems, expose other workers, and patients to COVID-19; but will also have a negative impact on the morale of professional colleagues.

## Ethics Statement

Written, informed consent was obtained from the individual/legal guardian/next of kin for the publication of any potentially identifiable images or data included in this article.

## Author Contributions

SB devised the project, the main conceptual ideas and proof outline, encouraged DS to investigate and supervised the findings of this work. SB and DS wrote the first draft of the manuscript. All authors discussed the results and recommendations and contributed to the final manuscript. The consortium would like to thank PJ for sharing an illustrative case example included in the study. The opinions expressed in this article are those of the authors and do not necessarily represent the decisions, official policy, or opinions of the affiliated institutions.

## Conflict of Interest

The authors declare that the research was conducted in the absence of any commercial or financial relationships that could be construed as a potential conflict of interest.
